# Direct reprogramming of epithelial cell rests of malassez into mesenchymal-like cells by epigenetic agents

**DOI:** 10.1038/s41598-020-79426-4

**Published:** 2021-01-20

**Authors:** Koki Yoshida, Osamu Uehara, Yoshihito Kurashige, Durga Paudel, Aya Onishi, Puja Neopane, Daichi Hiraki, Tetsuro Morikawa, Fumiya Harada, Rie Takai, Jun Sato, Masato Saitoh, Yoshihiro Abiko

**Affiliations:** 1 Division of Oral Medicine and Pathology, Department of Human Biology and Pathophysiology, School of Dentistry, Health Sciences University of Hokkaido, 1757 Kanazawa, Ishikari-Tobetsu, Hokkaido 061-0293 Japan; 2 Division of Disease Control and Molecular Epidemiology, Department of Oral Growth and Development, School of Dentistry, Health Sciences University of Hokkaido, 1757 Kanazawa, Ishikari-Tobetsu, Hokkaido 061-0293 Japan; 3 Division of Pediatric Dentistry, School of Dentistry, Health Sciences University of Hokkaido, 1757 Kanazawa, Ishikari-Tobetsu, Hokkaido 061-0293 Japan; 4 Division of Reconstructive Surgery for Oral and Maxillofacial Region, Department of Human Biology and Pathophysiology, School of Dentistry, Health Sciences University of Hokkaido, 1757 Kanazawa, Ishikari-Tobetsu, Hokkaido 061-0293 Japan; 5 Division of Oral and Maxillofacial Surgery, Department of Human Biology and Pathophysiology, School of Dentistry, Health Sciences University of Hokkaido, 1757 Kanazawa, Ishikari-Tobetsu, Hokkaido 061-0293 Japan; 6 Research Institute of Health Sciences, Health Sciences University of Hokkaido, 1757 Kanazawa, Ishikari-Tobetsu, Hokkaido 061-0293 Japan

**Keywords:** Stem-cell differentiation, Histone analysis, Methylation analysis

## Abstract

The DNA demethylating agent, 5-Azacytidine (5Aza), and histone deacetylase inhibitor, valproic acid (Vpa), can improve the reprogramming efficiencies of pluripotent cells. This study aimed to examine the roles of 5Aza and Vpa in the dedifferentiation of epithelial cell rests of Malassez (ERM) into stem-like cells. Additionally, the ability of stem-like cells to differentiate into mesenchymal cells was evaluated. ERM was cultured in embryonic stem cell medium (ESCM) with 1 µM of 5Aza, or 2 mM of Vpa, or a combination of 5Aza and Vpa. The cells stimulated with both 5Aza and Vpa were named as progenitor-dedifferentiated into stem-like cells (Pro-DSLCs). The Pro-DSLCs cultured in ESCM alone for another week were named as DSLCs. The stem cell markers were significantly higher in the DSLCs than the controls (no additions). The mRNA and protein levels of the endothelial, mesenchymal stem, and osteogenic cell markers were significantly higher in the Pro-DSLCs and DSLCs than the controls. The combination of a demethylating agent and a deacetylated inhibitor induced the dedifferentiation of ERM into DSLCs. The Pro-DSLCs derived from ERM can be directly reprogrammed into mesenchymal-like cells without dedifferentiation into stem-like cells. Isolated ERM treated with epigenetic agents may be used for periodontal regeneration.

## Introduction

Stem cells/progenitor cells have been isolated for use in regenerative dentistry. Several methods have been employed to purify the cells from human exfoliated deciduous teeth^1^. However, it is difficult to obtain cells of adequate quality and numbers; thus, a stable supply must be generated for their application in regenerative dentistry. Since the development of induced pluripotent stem (iPS) cells, several cells derived from dental cells and tissues such as the apical papillae, dental pulp, primary teeth, third molars, oral mucosa, and gingiva have been reprogrammed by transduction with Yamanaka factors, which include the Octamer-binding transcription factor 3/4 (*Oct3/4*), SRY-box transcription factor 2 (*Sox2*), Kruppel like factor 4 (*Klf4*), and *c-Myc*^2–4^. Epigenetics is the study of changes in gene expression that do not involve alterations in the gene sequences. Epigenetic alterations have been observed during the development of iPS cells^5^. Furthermore, the generation of reprogramming cells by inducing epigenetic alterations has been attempted recently^6–10^. DNA methylation and deacetylation are two major mechanisms of epigenetic modifications. The DNA demethylating agent (DNA methyltransferase inhibitor; DNMTi), 5-Azacytidine (5Aza), may favor reprogramming of fibroblasts, which is insufficient in the transition to pluripotency^6^. Moreover, 5Aza and valproic acid (Vpa), a histone deacetylase inhibitor (HDACi), have been reported to improve the reprogramming efficiencies of pluripotent stem cells^7^. Recently, low concentrations of HDAC were used to promote the dedifferentiation of dental pulpal stem cells^11^. However, epigenetic agents have not been used on other cells derived from dental tissues.

The expression of *Oct3/4*, *Sox2*, and *Myc* has been observed in the cells of the dental papilla and follicle^12^. *Sox2* was localized in the dental lamina but not in the epithelial cell rests of Malassez (ERM)^13^. Cultured epithelial cells derived from the ERM possess stem cell properties and are capable of epithelial–mesenchymal transitions^14,15^. Therefore, we hypothesized that epigenetic modifications might induce the dedifferentiation of ERM with a potential for reprogramming the cells. The ERM is thought to contribute to periodontal regeneration by repairing the cementum and forming the mineralized tissue on the root surface^16^; thus, we presumed that the dedifferentiated ERM could be used for periodontal regeneration. In the present study, we examined the roles of the DNMTi and HDACi in dedifferentiating ERM into stem cell-like cells. In addition, the ability of these stem cell-like cells to differentiate into mesenchymal-like cells, such as endothelial, mesenchymal stem, and osteogenic cells, that constitute the periodontal ligament was evaluated.

## Results

**Figure 1:**
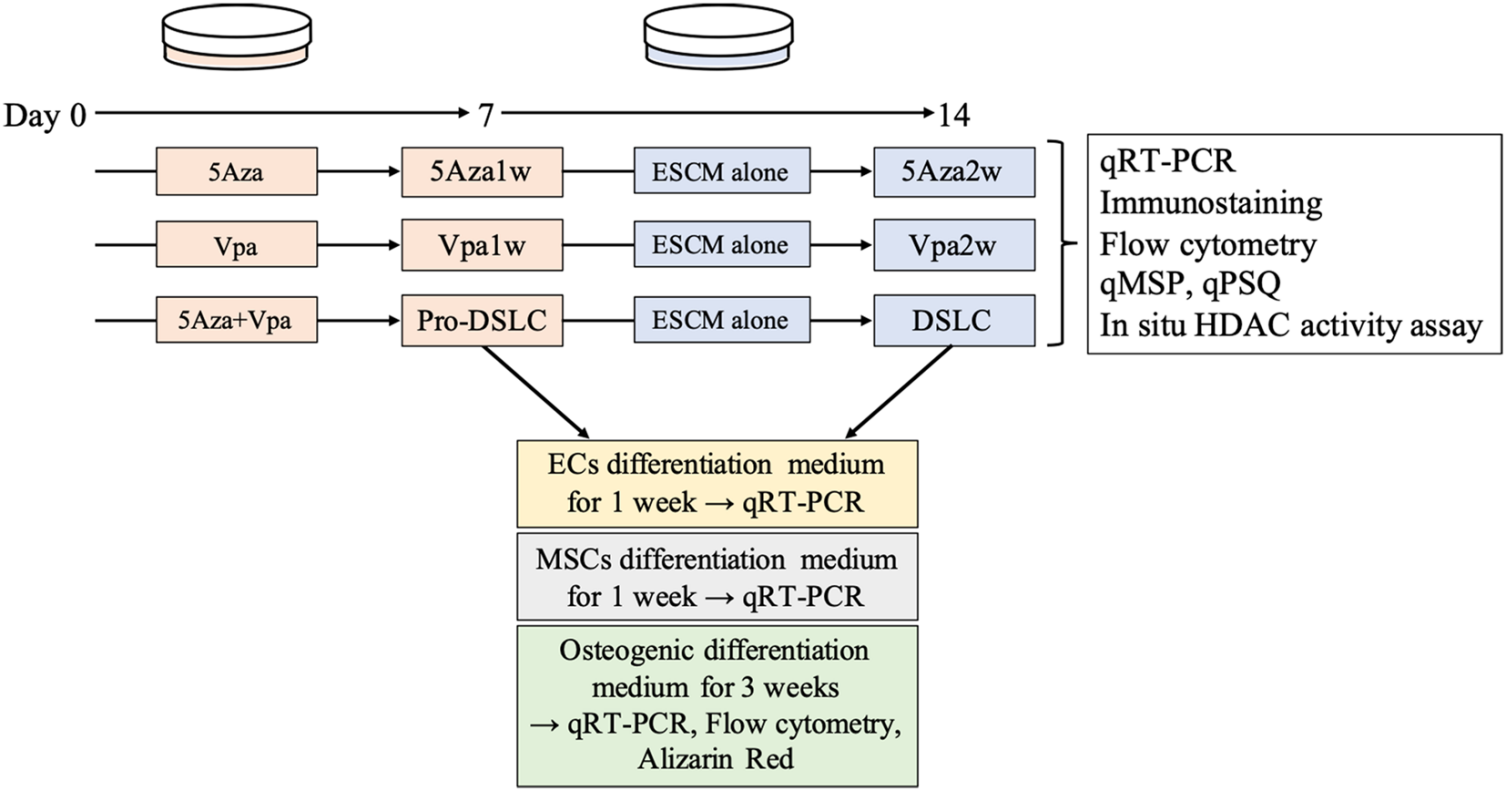
Schematic diagram of the whole experiment. The ERM was cultured in ESCM using culture dishes under the following conditions: 1 µM of 5Aza; and/or 2 mM of Vpa; and no additions (control) for 1 week. The cells stimulated with 5Aza alone and Vpa alone were named as 5Aza1w and Vpa1w, respectively. The cells stimulated with both 5Aza and Vpa were named as a progenitor-dedifferentiated into stem-like cells (Pro-DSLCs). Some of the cells were cultured in ESCM alone for another week. The 5Aza1w and Vpa1w cultured in ESCM alone for another week were named as 5Aza2w and Vpa2w, respectively. The Pro-DSLCs cultured in ESCM alone for another week were named as a dedifferentiated into stem-like cells (DSLCs). The controls (ERM cells), Pro-DSLCs, and DSLCs were cultured in Endothelial cells (ECs) differentiation medium for 1 week, bone marrow mesenchymal stem cells (MSCs) differentiation medium for 1 week, and osteogenic differentiation medium for 3 weeks.

The ERM was cultured in Embryonic Stem Cell Medium (ESCM) using culture dishes under the following conditions: 1 µM of 5Aza; and/or 2 mM of Vpa; and no additions (control) for 1 week. The cells stimulated with 5Aza alone and Vpa alone were named as 5Aza1w and Vpa1w, respectively. The cells stimulated with both 5Aza and Vpa were named as a progenitor-dedifferentiated into stem-like cells (Pro-DSLCs). Some of the cells were cultured in ESCM alone for another week. The 5Aza1w and Vpa1w cultured in ESCM alone for another week were named as 5Aza2w and Vpa2w, respectively. The Pro-DSLCs cultured in ESCM alone for another week were named as dedifferentiated into stem-like cells (DSLCs). The controls (ERM cells), Pro-DSLCs, and DSLCs were further cultured in Endothelial cells (ECs) differentiation medium for 1 week, bone marrow mesenchymal stem cells (MSCs) differentiation medium for 1 week, and osteogenic differentiation medium for 3 weeks (Fig. 1).

**Figure 2:**
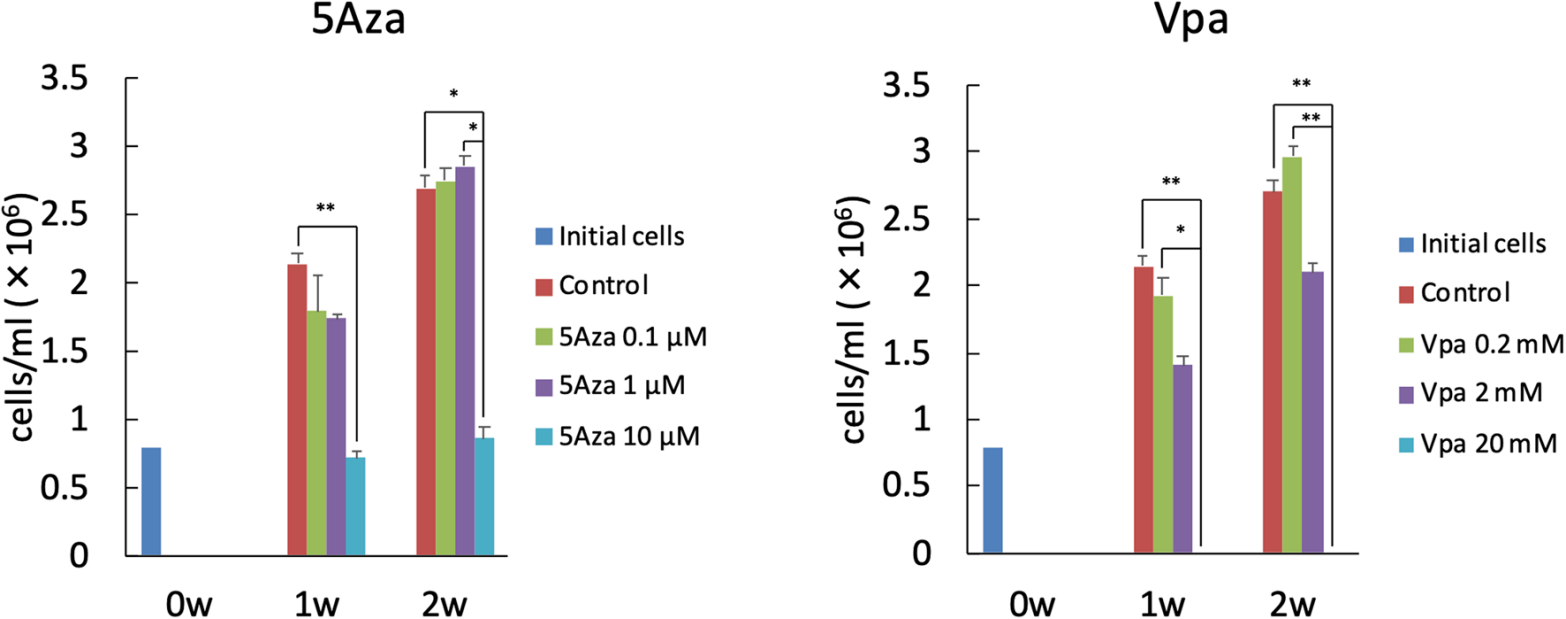
Cell viability. The ERM was cultured at a cell density of 0.8 × 10^6^ cells/ml (initial cells) in ESCM using 60 mm dishes under the following conditions: 0.1, 1 or 10 µM of 5Aza; and 0.2, 2 or 20 mM of Vpa; and no additions (control) for 1 week. Some of the cells were cultured in ESCM alone for another week. The numbers of viable ERM were significantly decreased in the groups stimulated with 10 µM of 5Aza when compared to those in the controls at week 1, and those in the controls and 1 µM of 5Aza at week 2 (**p < 0.01, *p < 0.05; Kruskal–Wallis test, n = 5). The numbers of viable ERM were significantly decreased in the groups stimulated with 20 mM of Vpa at weeks 1 and 2 when compared to those in the controls and 0.2 mM of Vpa. No significant differences in cell numbers were observed between any of the other conditions and the controls.

The cytotoxicities of different concentrations of 5Aza and Vpa were evaluated to determine the optimal concentrations. The numbers of viable ERM were significantly decreased in the groups stimulated with 10 µM of 5Aza when compared to those in the controls (no additions) at week 1, and those in the controls and 1 µM of 5Aza at week 2 (*p* < 0.01, 0.05; Fig. 2). The numbers of viable ERM were significantly decreased in the groups stimulated with 20 mM of Vpa at weeks 1 and 2 when compared to those in the controls (no additions) and 0.2 mM of Vpa. No significant differences in cell numbers were observed between any of the other conditions and the controls. Consequently, we decided to use 1 µM and 2 mM of 5Aza and Vpa, respectively, for further experiments.

**Figure 3:**
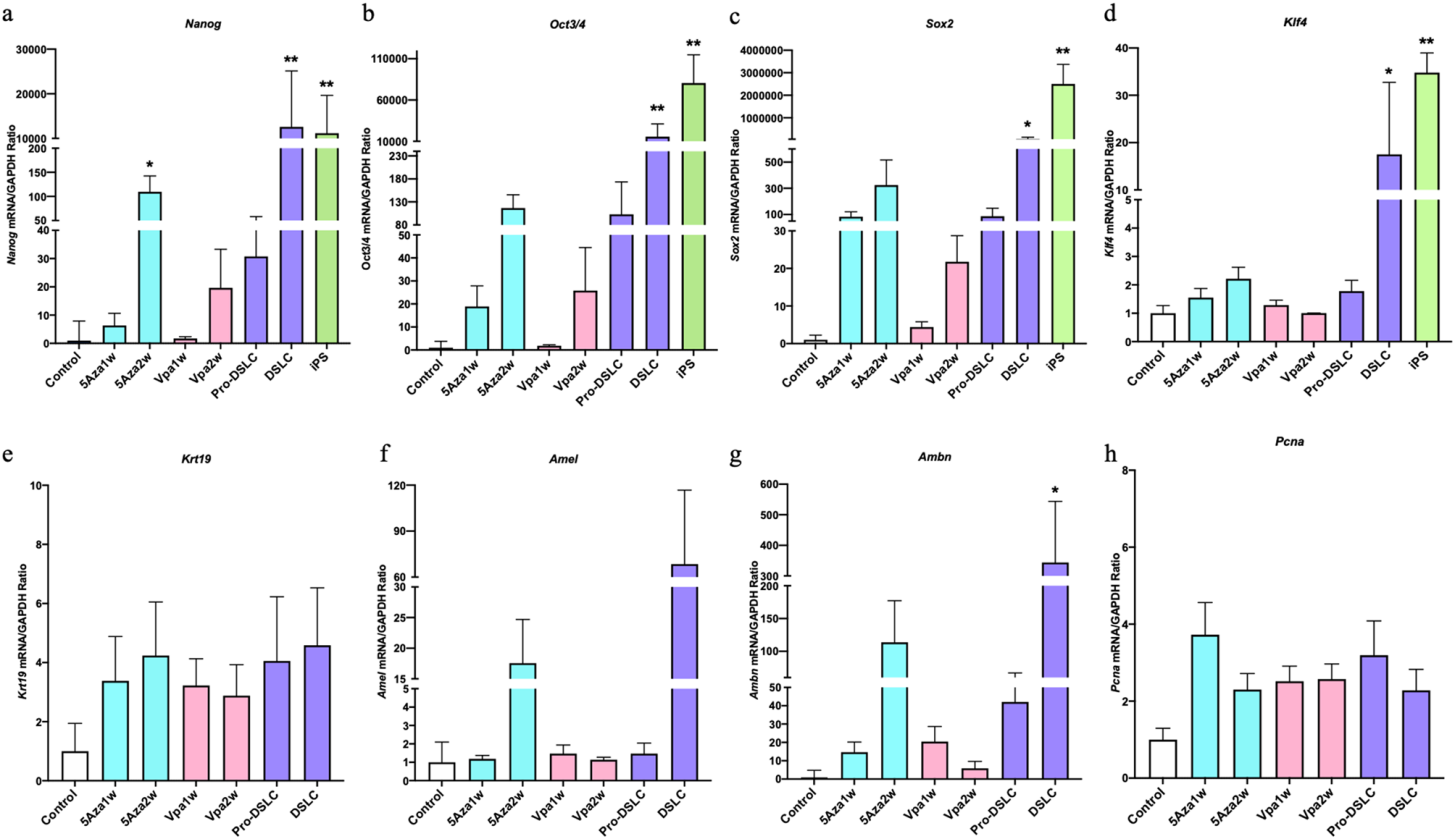
Bar graphs showing the results of the quantitative real-time reverse transcription polymerase chain reaction (qRT-PCR) in the control (ERM cells) and experimental cells. The expression levels of all the stem cell markers, including *Nanog*, *Oct3/4*, *Sox2*, and *Klf4* were significantly higher in the DSLCs and iPS cells when compared to those in the controls (**p < 0.01, *p < 0.05; Kruskal–Wallis test, n = 5; **a**–**d**). No significant increases in the expression levels of the odontogenic markers, except for *Ambn*, were observed in the DSLCs (e–g). In the cells treated with 5Aza alone for 1 week and followed by ESCM alone for another week (5Aza2w), the expression levels of *Nanog* were significantly upregulated (*p < 0.05; **a**–**g**). No significant increase in the expression level of any of the markers was noted in the Pro-DSLCs, the cells treated with 5Aza alone for 1 week (5Aza1w), the cells treated with Vpa alone for 1 week (Vpa1w), and the cells treated with Vpa alone for 1 week and followed by ESCM alone for another week (Vpa2w; **a**–**g**). No significant increase in the expression level of the tumorigenic marker, *Pcna*, was observed under any of the culture conditions (**h**).

We determined the mRNA expression levels of the stem cell markers Nanog homeobox (*Nanog*), *Oct3/4*, *Sox2*, and *Klf4*, odontogenic markers amelogenin (*Amel*), ameloblastin (*Ambn*), and keratin19 (*Krt19*), and tumorigenic marker proliferating cell nuclear antigen (*Pcna*). iPS cells were used as positive controls. The expression levels of all the stem cell markers, *Nanog*, *Oct3/4*, *Sox2*, and *Klf4*, following stimulation with 1 µM of 5Aza and/or 2 mM of Vpa, were significantly higher in the dedifferentiated into stem-like cells (DSLCs) and iPS cells when compared to those in the controls (ERM cells) (*p* < 0.01, 0.05; Fig. 3a–d). No significant increases in the expression levels of the odontogenic markers, except for ameloblastin (*Ambn*), were observed in the DSLCs (Fig. 3e–g). The expression levels of *Nanog* were significantly upregulated in the cells treated with 5Aza alone for 1 week and followed by ESCM alone for another week (5Aza2w; *p* < 0.05; Fig. 3a–g). No significant increase in the expression level of any of the markers was noted in the progenitor-DSLCs (Pro-DSLCs), the cells treated with 5Aza alone for 1 week (5Aza1w), the cells treated with Vpa alone for 1 week (Vpa1w), and the cells treated with Vpa alone for 1 week and followed by ESCM alone for another week (Vpa2w; Fig. 3a–g). No significant increase in the expression level of *Pcna* was observed under any of the culture conditions (Fig. 3h).

**Figure 4:**
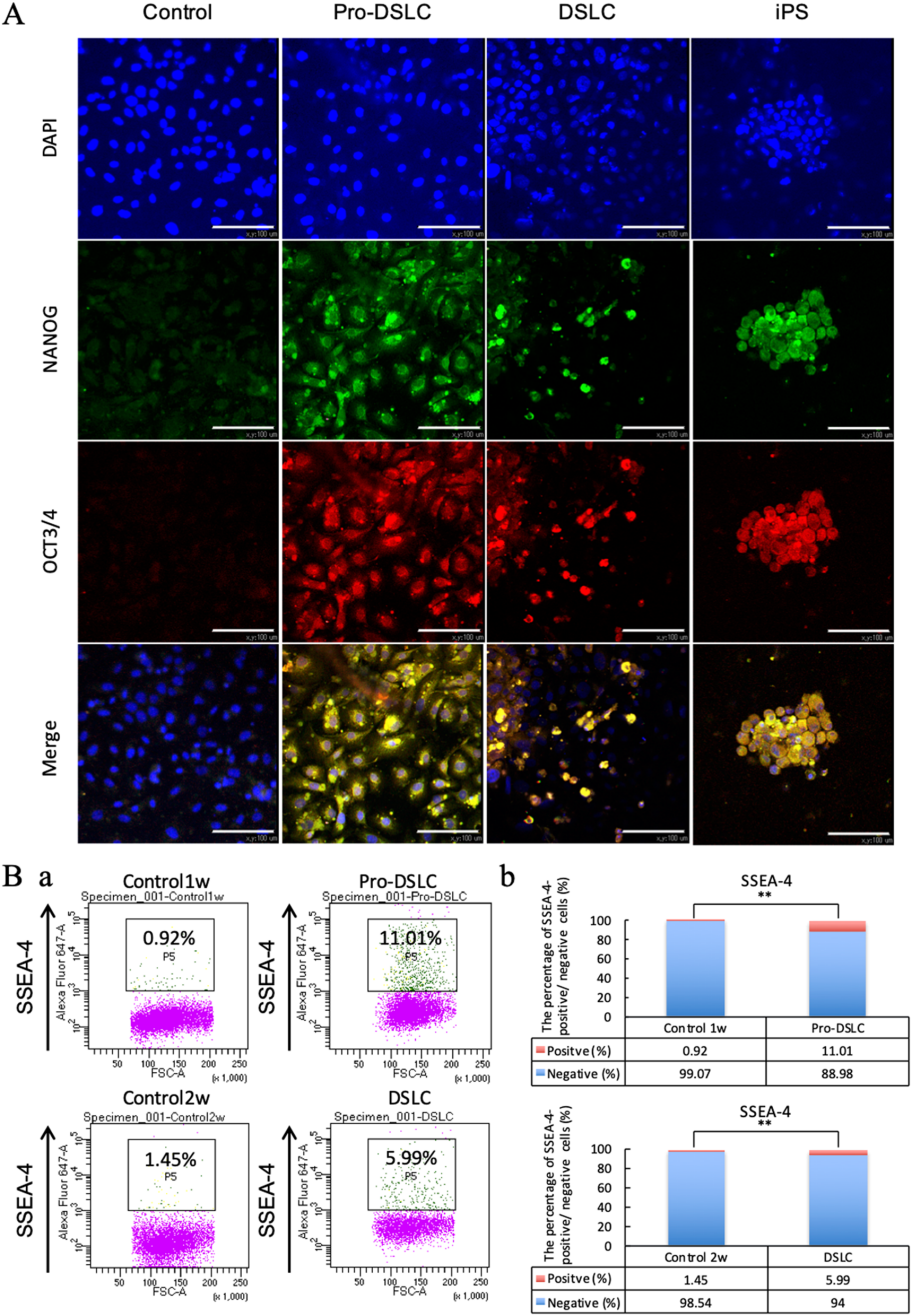
Graphs showing the results of the immunofluorescence (**A**) and flow cytometry (**B**) tests in the control (ERM cells) and experimental cells. The Pro-DSLCs and DSLCs showed positive staining for NANOG and OCT3/4 (**A**). Merge, nuclear visualization (blue), NANOG (green), and OCT3/4 (red) visualization. Scale bar = 100 µm. Magnification × 400. The controls, Pro-DSLCs, and DSLCs were stained with SSEA-4 (**B**). The percentage of SSEA-4-positive cells among the Pro-DSLCs, and DSLCs was significantly higher than that in the controls (**p < 0.01; χ^2^ tests, n = 5). Gating the SSEA-4-positive cells by flow cytometry (**Ba**). Analysis of the percentage of SSEA-4-positive/ negative cells (**Bb**).

The expression levels of NANOG and OCT3/4 were ascertained at the protein level in the Pro-DSLCs and DSLCs via immunocytochemistry; both cells showed positive staining for the two markers. iPS cells were used as positive controls (Fig. 4A). The percentage of SSEA-4-positive cells in the Pro-DSLCs and DSLCSs was significantly higher than that in the controls (ERM cells) (*p* < 0.01; Fig. 4Ba,b).

**Figure 5:**
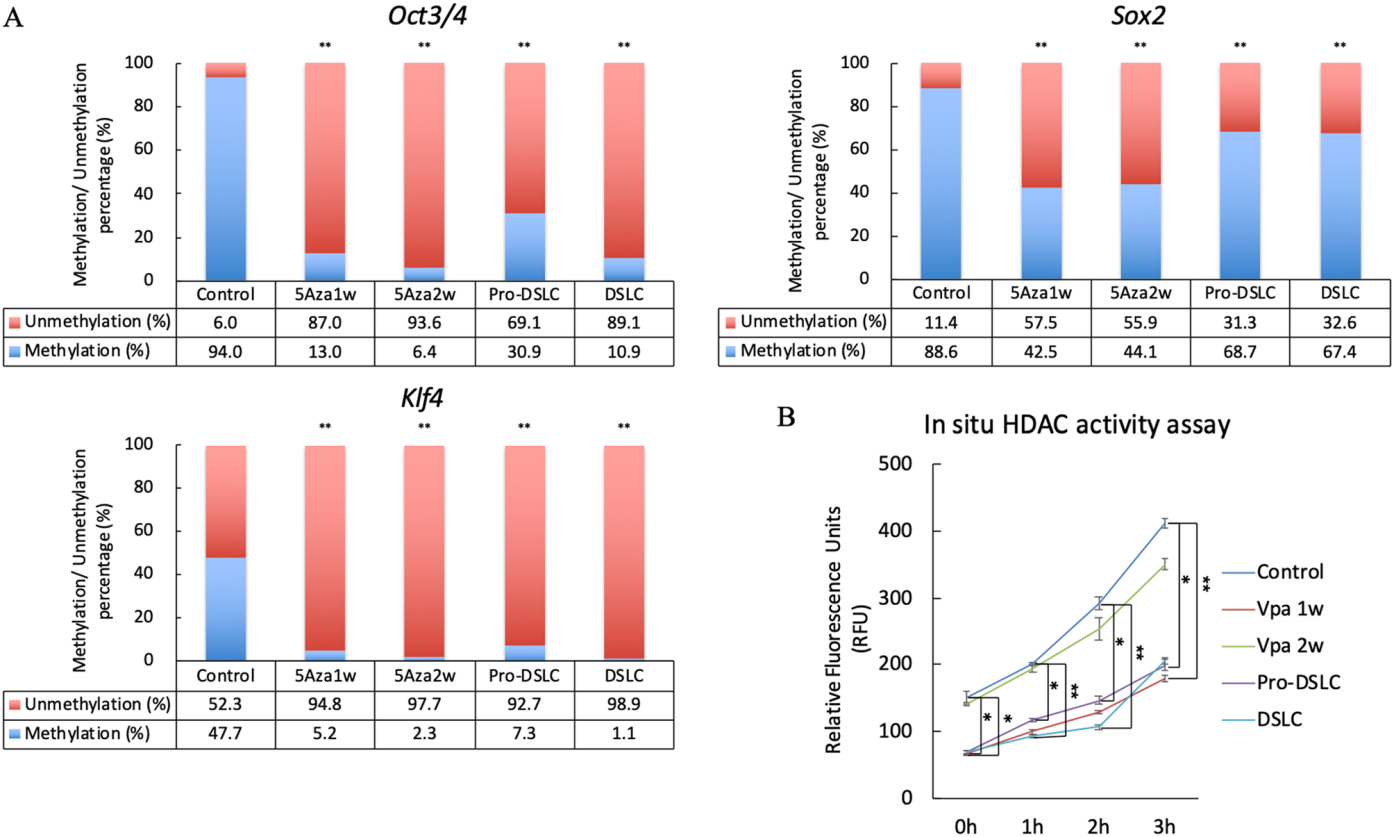
Results of the quantitative methylation-specific PCR (qMSP) (**A**) and in situ HDAC activity assay (**B**). DNA methylation of *Oct3/4*, *Sox2*, and *Klf4* was analyzed using qMSP. The DNA methylation percentage levels of *Oct3/4*, *Sox2*, and *Klf4* in the cells treated with 5Aza alone (5Aza1w, and 5Aza2w), Pro-DSLCs, and DSLCs were significantly lower than those in the controls (ERM cells) (**p < 0.01; χ^2^ tests, n = 5). Each sample showed an increase in HDAC activity in a time-dependent manner (B). HDAC activities were significantly lower in the Pro-DSLCs, DSLCs, and the cells treated with Vpa alone for 1 week (Vpa1w) when compared to those in the controls (ERM cells) (**p < 0.01, *p < 0.05; Kruskal–Wallis test, n = 5).

Quantitative methylation-specific PCR analysis (qMSP) revealed that the DNA methylation percentage levels of *Oct3/4*, *Sox2*, and *Klf4* in the cells treated with 5Aza alone (5Aza1w, and 5Aza2w), Pro-DSLCs, and DSLCs were significantly lower than those in the controls (ERM cells) (*p* < 0.01; Fig. 5A). Furthermore, the cells in each group of Vpa1w, Vpa2w, Pro-DSLCs, DSLCs and the controls (ERM cells) showed increased HDAC activity in a time-dependent manner. The HDAC activities in the Pro-DSLCs, DSLCs, and Vpa1w were significantly lower than those in the controls (ERM cells) (*p* < 0.01, 0.05; Fig. 5B). In order to verify the reliability of the MSP data, DNA methylation analysis was performed at the CpG sites using the quantitative pyrosequencing analysis (qPSQ). The DNA methylation percentage levels of the CpG sites in *Oct3/4* and *Sox2* in the cells treated with 5Aza alone (5Aza1w, and 5Aza2w), Pro-DSLCs, and DSLCs were significantly lower than those in the controls (ERM cells) (*p* < 0.01; Supplementary Fig. S1).

**Figure 6:**
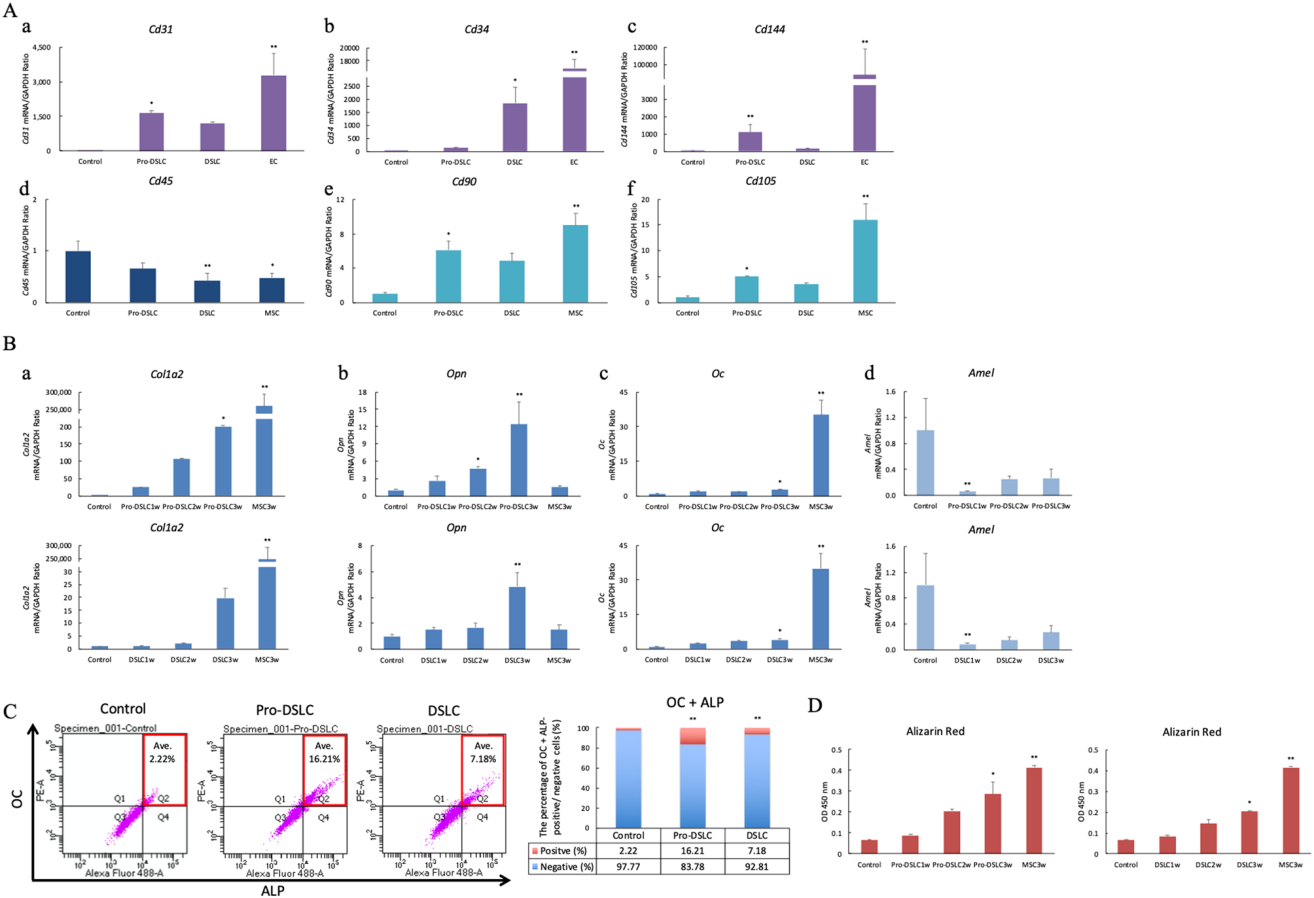
Graphs showing the differentiation of controls (ERM cells), Pro-DSLCs, and DSLCs into ECs, MSCs, and osteoblasts. The expression levels of *Cd31* and *Cd144* (endothelial cell markers) in Pro-DSLCs and ECs, and *Cd34* (endothelial cell marker) in DSLCs and ECs cultured in the endothelial differentiation medium were significantly higher than those in the controls (**p < 0.01, *p < 0.05; Kruskal–Wallis test, n = 5; **Aa**–**c**). The expression levels of *Cd45* (mesenchymal stem cell-negative marker) in the DSLCs and MSCs were significantly lower than those in the controls (**p < 0.01, *p < 0.05; Kruskal–Wallis test, n = 5; **Ad**). The expression levels of *Cd90* and *Cd105* (mesenchymal stem cell-positive markers) in the Pro-DSLCs and MSCs were significantly higher than those in the controls (**p < 0.01, *p < 0.05; **Ae**,**f**). The mRNA expression levels of osteogenic markers *Col1a2* in Pro-DSLCs and MSCs, *Opn* in Pro-DSLCs and DSLCs, and *Oc* in Pro-DSLCs, DSLCs and MSCs, cultured in osteogenic differentiation culture medium were significantly higher than those in the controls (**p < 0.01, *p < 0.05; Kruskal–Wallis test, n = 5; **Ba**–**c**). Conversely, the significantly decreased mRNA levels of *Amel* (odontogenic marker) were observed in the Pro-DSLCs and DSLCs compared to those in the controls (**p < 0.01; **Bd**). The expression of OC and ALP was evaluated using a flow cytometer (**C**). Flow cytometry revealed a significant increase in the number of cells stained with OC and ALP in the Pro-DSLCs and DSLCs when compared to those in the controls (**p < 0.01; χ^2^ tests, n = 5). The calcification levels determined by Alizarin Red were significantly higher in the Pro-DSLCs, DSLCs, and MSCs than those in the controls (**p < 0.01, *p < 0.05; Kruskal–Wallis test, n = 5; **D**).

The controls (ERM cells), Pro-DSLCs, and DSLCs were cultured in ECs differentiation medium for 1 week, MSCs differentiation medium for 1 week, and osteogenic differentiation medium for 3 weeks. The mRNA expression levels of the endothelial cell markers platelet and endothelial cell adhesion molecule 1 (*Cd31*), C34 molecule (*Cd34*), and cadherin 5 (*Cd144*), mesenchymal stem cell-negative marker protein tyrosine phosphatase receptor type C (*Cd45*), mesenchymal stem cell-positive markers Thy-1 cell surface antigen (*Cd90*), and endoglin (*Cd105*); osteogenic markers collagen type I alpha 2 chain (*Col1a2*), osteopontin (*Opn*), and osteocalcin (*Oc*), and ameloblastic marker *Amel* were evaluated. ECs and MSCs were used as positive controls. The expression levels of *Cd31* and *Cd144* in Pro-DSLCs and ECs, and *Cd34* in DSLCs and ECs, cultured in the endothelial differentiation medium were significantly higher than those in the controls (*p* < 0.01, 0.05; Fig. 6Aa–c). The expression levels of *Cd45* in the DSLCs and MSCs cultured in MSCs differentiation culture medium were significantly lower than those in the controls (*p* < 0.01, 0.05; Fig. 6Ad). The expression levels of *Cd90*, and *Cd105* in the Pro-DSLCs and MSCs were significantly higher than those in the controls (*p* < 0.01, 0.05; Fig. 6Ae,f).The mRNA expression levels of *Col1a2* in Pro-DSLCs and MSCs, *Opn* in Pro-DSLCs and DSLCs, and *Oc* in Pro-DSLCs, DSLCs and MSCs, cultured in osteogenic differentiation culture medium were significantly higher than those in the controls (*p* < 0.01, 0.05; Fig. 6Ba–c); alternatively, a significant decrease in the mRNA levels of *Amel* were observed in the Pro-DSLCs and DSLCs when compared to those in the controls (*p* < 0.01; Fig. 6Bd). Flow cytometry revealed a significant increase in the number of cells stained with OC and alkaline phosphatase (ALP) in the Pro-DSLCs and DSLCs when compared to those in the controls (*p* < 0.01; Fig. 6C). The Pro-DSLCs, DSLC, and MSCs showed increased positive staining for Alizarin Red in a time-dependent manner (Supplementary Fig. S2). The calcification levels determined by Alizarin Red staining in the Pro-DSLCs, DSLCs, and MSCs were significantly higher than those in the controls (*p* < 0.01, 0.05; Fig. 6D).

## Discussion

In the present study, epigenetic agents were shown to stimulate the transition of ERM into stem cell-like cells. The expression of stem cell markers was significantly upregulated in ERM cultured in ESCM for an additional week after stimulation with the combination of 5Aza and Vpa (DSLCs). The DSLCs derived from the epithelial cells were able to differentiate into mesenchymal-like cells and exhibit increased expression levels of each of the mesenchymal differentiation markers under different culture conditions. Similarly, the Pro-DSLCs were also differentiated into mesenchymal-like cells under the various culture conditions. These findings indicated that ERM could be stimulated for dedifferentiation into stem cell-like cells via both demethylation and deacetylase inhibition. The differentiation of Pro-DSLCs into mesenchymal-like cells, despite no significant increases in the mRNA expression levels of any of the stem cell markers, may be attributed to direct reprogramming. Positive stainings for NANOG and OCT3/4 were observed in the Pro-DSLCs. The cells that were positively stained with NANOG and OCT3/4 did not present with high mRNAs levels of the corresponding genes as seen in the case of the iPS or stem cells^17^, which is termed as highly permissive state cells^18^. Highly permissive cells are generally observed during the process of direct reprogramming. The Pro-DSLCs might represent a type of the highly permissive state cells and play a role in direct reprogramming. The ERM cells were cultured in ESCM for an additional week after the cells were stimulated with the epigenetic agents to generate DSLCs, as described previously^8^. Pro-DSLCs can be reprogrammed into mesenchymal cells; therefore, culture period in ESCM alone after epigenetic stimulation may not be required for ERM and some other cells to induce direct reprogramming of the cells into other cells. Further investigations are required to confirm this speculation.

Several genes have been epigenetically expressed in iPS cells^5^. Consequently, epigenetic agents, such as DNMTi and HDACi have been used to generate and improve the production of reprogramming cells^6–10^. Fibroblasts have been mainly used as an original cell for the generation of iPS and reprogramming cells^19^. Although the reprogramming efficiency of fibroblasts transducted with Yamanaka factors was improved by the DNMTi and HDACi, the cells could not be completely generated into stem cells without the Yamanaka factors^20^. The DSLCs in the current study expressed *Ambn* and other stem cell markers, indicating that these cells were not completely transformed into stem cells, unlike the iPS cells. Thus, the use of epigenetic agents alone may not be sufficient to generate stem cells. In previous studies, fibroblasts were directly reprogrammed into insulin-secreting cells by epigenetic agents in the absence of the Yamanaka factor^17,20^. Both DSLCs and Pro-DSLCs could be differentiated into other cell types in the present study. The epigenetic agents may be involved in the direct reprogramming of the cells into other cell types via pleiotropic stem-like cells. Both DSLCs and Pro-DSLCs originated from ERM, a type of keratinocyte. The efficiencies of the cells that are transformed into pleiotropic stem-like cells may not matter if they are originally derived from fibroblasts or epithelial cells. The efficiencies of the iPS cells generated from primary keratinocytes have been reported to be less than that of cells derived from fibroblasts^19^. In another study, the efficiency of reprogramming was higher in immature cells when compared to that in the mature cells^21^. According to some studies, ERM cells are immature with stem cell-like features and properties^15,22^. Hence, they may have a higher potential to generate pluripotent stem cells than keratinocytes derived from other types of stratified squamous epithelium. Cell populations contain less than 1% of side population (SP) cells^23,24^; therefore, the ERM might contain only a small number of SP cells. In fact, the positive staining for both NANOG and OCT3/4 was hardly observed in the ERM (controls). The SP cells derived from tumor cells had highly growth potentials and could be inhibited by either DNMTi or HDACi ^25,26^. The small number of SP cells might not grow as a part of DSLCs and Pro-DSLCs. The use of specific media for the differentiation of ES or iPS cells into ECs^27,28^, MSCs^29^, and osteogenic cells^30^ have been reported. In the present study, we used the same media as those reported in the afore-mentioned studies, and each one had induced the differentiation of Pro-DSLCs and DSLCs into mesenchymal-like cells. The expression levels for the osteogenic markers in these mesenchymal-like cells were relatively weak as compared to the positive controls. The expression levels of *Opn* in Pro-DSLCs and DSLC were higher than those in the positive controls, whereas the expression levels of *Oc* in Pro-DSLCs and DSLC were lower than those in the positive controls. The calcification levels in Pro-DSLCs and DSLC were lower than those in the positive controls. Early mature osteoblasts express *Opn,* and the expression of *Oc* was upregulated during osteoblast maturation^31^. The differences in the expression level of osteogenic markers between the mesenchymal-like cells and the positive controls may depend on the level of osteoblastic differentiation. Further investigations are needed to determine more optimal conditions for their mesenchymal differentiations.

In conclusion, the present study demonstrated that the combination of a demethylating agent and a deacetylated inhibitor induced the dedifferentiation of ERM into stem cell-like cells. The progenitor stem-like cells derived from ERM can be directly reprogrammed into mesenchymal-like cells without dedifferentiation into stem-like cells. Isolated ERM may be used for periodontal regeneration because they can be differentiated into several cells that mainly constitute the periodontal tissues. However, further investigations are needed to prove this hypothesis.

## Materials and methods

Figure 1 shows a schematic diagram of the experiment in the current study (Fig. 1).

### Culture of epithelial cells from porcine ERM

Epithelial cells were isolated from porcine ERM and cultured using the method described by Brunette et al., and Kurashige et al^32,33^. Six month-old porcine jaws were obtained from the livestock sales division (HOKUREN Federation of Agricultural Cooperatives, Hokkaido, Japan) and transported to our laboratory on ice. Porcine primary premolars were extracted and explants of the periodontal ligament were cultured in Dulbecco’s modified Eagle medium (Sigma-Aldrich, MO, USA) containing 10% fetal bovine serum (Gibco, Thermo Fisher Scientific, MA, USA) and 2% penicillin–streptomycin (Sigma-Aldrich) at 37 °C in an incubator supplied with 5% CO_2_. An outgrowth composed of both epithelial-like and fibroblast-like cells was observed. The cells that were more resistant to detachment by 10% dispase (Goudousyusei, Tokyo, Japan) were separated into epithelial cell populations and cultured. The cells that were less resistant to detachment were discarded. The separation procedure was repeated several times to remove any fibroblast cells.

### Cell viability

The ERM cells were cultured at a cell density of 0.8 × 10^6^ cells/ml in Primate Embryonic Stem Cell Medium (ESCM, ReproCELL, Kanagawa, Japan) containing 5 ng/ml basic fibroblast growth factor (ReproCELL) and 0.5% penicillin–streptomycin using 60 mm dishes under the following conditions: 0.1, 1 or 10 µM of 5Aza (Tokyo Chemical Industry, Tokyo, Japan); 0.2, 2 or 20 mM of Vpa (FUJIFILM Wako Pure Chemical, Osaka, Japan); and no additions (control) for 1 week. Some of the cells were cultured in ESCM alone for another week because ESCM is known to improve the embryonic stem (ES) property of the cells^34,35^. The cells were then collected from each culture system using 0.25% Trypsin–EDTA (Gibco, Thermo Fisher Scientific). The dead cells were stained with 0.5% trypan blue and the number of viable cells was counted using a hemocytometer.

### Quantitative real-time RT-PCR

We determined the mRNA expression levels of the stem cell markers *Nanog*, *Oct3/4*, *Sox2*, and *Klf4*, odontogenic markers amelogenin (*Amel*), *Ambn*, and keratin19 (*Krt19*), and tumorigenic marker *Pcna* in each ERM system treated with 1 µM of 5Aza alone for 1 week (5Aza1w), and followed by ESCM alone for another week (5Aza2w), 2 mM of Vpa alone for 1 week (Vpa1w), and followed by ESCM alone for another week (Vpa2w), and the combination of both 5Aza (1 µM) and Vpa (2 mM) for 1 week (Pro-DSLCs), and followed by ESCM alone for another week (DSLCs; Fig. 1). Human iPS cells (253G1) provided by the RIKEN BRC through the National BioResource Project of the MEXT/AMED (Ibaraki, Japan) were used as positive controls for the stem cell markers^36^. Total RNA was extracted from the cells via the acid guanidine thiocyanate/phenol–chloroform method using TRizol (Invitrogen, Thermo Fisher Scientific MA, USA). One microgram of the RNA sample was reverse-transcribed (SuperScript reverse transcriptase, Invitrogen) according to the manufacturer’s instructions using oligo (dT) 12–18 primers (Invitrogen, Thermo Fisher Scientific). For quantitative real-time reverse transcription polymerase chain reaction (qRT-PCR), a reaction mixture was prepared using the KAPA SYBR Fast qPCR Kit (Kapa Biosystems, Roche, Basel, Switzerland), primers, and RT products. The primer sequences used in this study are shown in Table 1. The qRT-PCR was performed using Light Cycler Nano (Roche). The qPCR conditions included an initial incubation at 50 °C for 2 min, denaturation at 95 °C for 10 min, 45 cycles of denaturation at 95 °C for 15 s, and annealing at 55 °C for 1 min. The relative expression level of each mRNA was calculated using the ΔΔCq method^37^. Data are shown as a ratio of the target mRNA to the glyceraldehyde-3-phosphate dehydrogenase (*Gapdh*) mRNA.

**Table 1:** Primers for qRT-PCR, and qMSP.

Gene	Forward (5′–3′)	Reverse (5′-3′)	NCBI reference sequence
*Gapdh*	GTCGGTTGTGGATCTGACCT	TTGACGAAGTGGTCGTTGAG	NM_001206359.1, NM_002046.7
*Nanog*	TGAGGTTTATGGGCCTGAAG	TGGGACCTTTTCCTCCTTCT	NM_001129971.1, NM_024865.4
*Oct3/4*	CGAGGAGTCCCAGGACATCAA	ACTGAGCTGCAAAGCCTCAA	NM_001113060.1, NM_002701.6
*Sox2*	AACCCCAAGATGCACAACTC	CGGGGCCGGTATTTATAATC	NM_001123197.1, NM_003106.4
*Klf4*	AACCCCAAGATGCACAACTC	GTGGTCCGACCTTGAAAATG	NM_001031782.2, NM_001314052.2
*Amel*	CCCTTGCTATGCCTCTACCA	ACCACGGGTATGATTTGGTG	NM_213800.1
*Ambn*	CCTCAGCCTCCAATTTACCA	TCAAACGGGCTATTGGAAAC	NM_214037.1
*Krt19*	CTCACCATGCAGAACCTCAA	AGGTCCTCGATGGTCTTGAA	XM_003131437.3
*Pcna*	AACCTGCAGAGCATGGACTC	ATGTCTTCATTGCCAGCACA	NM_001291925.1
*Cd31*	CATTTCCAAAGTCAGCAGCA	ATCATCATGCCTCCCTTCTG	NM_213907.1
*Cd34*	GGAAACCACACCAGATGCTT	AGGTCTGAGGCTGGACAGAA	NM_214086.1
*Cd144*	GCAAAAATCTGGACCGAGAG	TGTGTACCTGGTCTGGGTGA	NM_001001649.2
*Cd45*	GCGCCAAGCAAAGTCAGAAA	CGGTAGGAGGCGTACAAGTC	XM_003130596.6
*Cd90*	TACCACCAACCTGCCCATTC	AGAAGTTGGTTCGAGAGCGG	NM_001146129.1
*Cd105*	CCATGCTGTCGTAGCAACCC	GGACCTCTTCTGTTCTCGTGG	NM_214031.1
*Col1a2*	TGCTCAGCTTTGTGGATACG	CCTGGGATACCATCATCACC	NM_001243655.1
*Opn*	CTTGGACAGCCAAGAGAAGG	TGGCTGACTTTGGGATTTTC	NM_214023.1
*Oc*	TCACACTGCTTGCCCTACTG	AGCCCATGATCCAGGTAGC	NM_001164004.1
*Oct3/4-M*	GGGATATTTGGTTTTCGATTTC	CCACTCCGACCTAAAATACG	
*Oct3/4-U*	GGGATATTTGGTTTTTGATTTTGT	CCCACTCCAACCTAAAATACAC	
*Sox2-M*	TTCGTATGTATAATATGATGGAGACG	GTTCATAAACCGCTTAACTCGAT	
*Sox2-U*	TTGTATGTATAATATGATGGAGATGG	ACATTCATAAACCACTTAACTCAAT	
*Klf4-M*	TTTTTTTAGGCGGAATATTTTTTC	ATTACGAACCCTTAACCTAACGTC	
*Klf4-U*	TTTTTTAGGTGGAATATTTTTTTGG	CATTACAAACCCTTAACCTAACATC	

### Immunostaining

The controls, Pro-DSLCs, and DSLCs were fixed with 60% Acetone/ 40% methanol in a chamber slide (Lab-Tek Chamber Slide System, Nunc, Thermo Fisher Scientific) at room temperature for 15 min. The fixed cells were rinsed with PBS, three times at 10 min intervals. After pre-incubation in blocking buffer (5% normal goat serum, Dako, Glostrup, Denmark) at room temperature for 1 h, the cells were incubated overnight with the primary anti-human antibodies NANOG and OCT3/4 (1: 100; Human Pluripotent Stem Cell 3-Color Immunocytochemistry Kit, R&D Systems, MN, USA) at 4 °C, according to the manufacturer's instructions. The next day, the cells were rinsed alternately with PBST and PBS (3 times at 10 min intervals) and incubated with goat anti-rabbit IgG (H + L) Secondary Antibody Alexa Fluor 488 conjugate (1: 1000; Thermo Fisher Scientific) in the dark at room temperature for 1 h. After the cells were rinsed alternately with PBST and PBS (4 times at 10 min intervals), they were incubated with DAPI Fluoromount-G (SouthernBiotech, AL, USA) for nuclear visualization. iPS cells were used as positive controls for the NANOG and OCT3/4. The cells were observed using a confocal microscope (Nikon EZ-C1, Tokyo, Japan).

### Flow cytometry

Flow cytometry was used to analyze the cells stained with stage-specific embryonic antigen-4 (SSEA-4). The controls, Pro-DSLCs, and DSLCs were harvested from each cell system and filtered using a FALCON Cell Strainer (100 μm; Corning, NY, USA). The cells were incubated in the dark for 30 min at 4 °C with SSEA-4 Alexa Fluor 647 antibodies (Human Induced Pluripotent Stem Cell Analysis and Sorting Kit, BD Biosciences, NJ, USA), and further incubated for 10 min at 4 °C with 7-Amino-Actinomycin D (7-AAD, BD Biosciences). SSEA-4 was analyzed using a flow cytometer (BD FACSAriaIIIu, BD Biosciences).

### Quantitative methylation-specific PCR analysis

In order to confirm the DNA methylation levels of the cells in the experiments, genomic DNA was extracted from each cell culture system using the DNeasy Blood & Tissue Kit (Qiagen, Hilden, Germany). The DNA sample (500 ng) was treated with sodium bisulfite using the EpiTect Fast Bisulfite Kits (Qiagen). The DNA methylation of *Oct3/4*, *Sox2*, and *Klf4* was analyzed via SYBR green-based qMSP after checking for the presence of CpG islands around the promoter region in the UCSC Genome Browser (http://genome.ucsc.edu/index.html). Two sets of qMSP primers were designed using MethPrimer (http://www.urogene.org/methprimer/; Supplementary Figs. S3A, B)^38^; one for unmethylated and the other for methylated DNA sequences (Table 1). For the qMSP, the bisulfite-treated DNA template was mixed with KAPA SYBR Fast qPCR Kit and a pair of primers. The qMSP conditions included an initial incubation at 50 °C for 2 min, denaturation at 95 °C for 10 min, 50 cycles of denaturation at 95 °C for 15 s, and annealing at 55 °C for 1 min. The percentage of DNA methylation in the samples was estimated using the following equation:

$$Methylated\, DNA (\%)=\frac{M}{M+U}\times 100(\%)=\frac{1}{1+\frac{U}{M}}\times 100(\%)=\frac{1}{1+{2}^{(-\Delta Cq)}}\times 100(\%)$$

where M is the copy number of methylated DNA, U is the copy number of unmethylated DNA, and ΔCq = Cq_U_ − Cq_M_^39^.

### Quantitative pyrosequencing analysis

In order to verify the reliability of the MSP data, DNA methylation analysis was performed at the CpG site using the qPSQ method (ALLIANCE Biosystems, Osaka, Japan). The target region of *Oct3/4*, which contained five CpG sites, and of *Sox2*, which contained four CpG sites (sequence 1) and three CpG sites (sequence 2), were amplified using the forward primer and biotinylated reverse primer (PSQ Assay Design; Qiagen, Hilden, Germany; Table 2; Supplementary Fig. S3C). However, it was not possible to perform the analysis due to an error in the PCR primer design owing to the dense CG region in *Klf4*. Genomic DNA (200 ng) was modified by sodium bisulfite using the EZ DNA Methylation-Lighting kit (Zymo Research, CA, USA), according to the manufacturer’s instructions. Bisulfite-modified DNA was amplified in a 20-μl reaction volume containing the PCR pre-mixture (Enzynomics, Korea), 1 μl of Primer-S (10 pmol/μl), and 1 μl of biotinylated-Primer-As (10 pmol/μl). The PCR products were visualized on 2% agarose gel via ethidium bromide staining for verification.

**Table 2:** Primers for qPSQ.

Gene	Primer	Size (base pair)
***Oct3/4***		
Forward	5′-GGTTGATTTTAGGATTTGGTTGAGT-3’	135
Biotinylated-reverse	5′-ACCATCCCTCCCCAAAAATCATAA-3’	
Sequencing primer	5′-AAGGGTTTTTAGGTGGGTTA-3’	
***Sox2***		
Forward	5′-ATGATGGAGAAGGAGTTGAAGT-3’	194
Biotinylated-reverse	5′-ATCTTAAAATTCTCTTAAACCATCT-3’	
Sequencing primer-1	5′-GTTAGTAGTAAATTTTAGGGGG-3’	
Sequencing primer-2	5′-GGTAATTAGAAGAATAGTTTAG-3’	

A DNA template was prepared from 16–18 μl of the biotinylated PCR product using streptavidin Sepharose HP beads (Cytiva, GE Healthcare Life Sciences, MA, USA) and multichannel pipets according to the PSQ 96 sample preparation guide. Fifteen picomoles of the respective sequencing primer were added for analysis. The sequencing was performed on a PyroMark ID system (Qiagen) using the Pyro Gold reagents kit (Qiagen), according to the manufacturer’s instruction and without any further optimization. The methylation index (MtI) of each target region in each sample was calculated as the average value of mC/(mC + C) for all the examined CpGs. All experiments included samples without templates as negative control.

### In situ* HDAC activity assay*

The HDAC activity of the cells in the experiments was confirmed with the In Situ HDAC Activity Fluorometric Assay Kit (BioVision Mountainview, CA, USA). Fluorescence signals were detected following excitation at 380 nm and emission at 450 nm using a fluorescence microplate reader (Infinite F200, TECAN, Männedorf, Switzerland).

### Differentiation of the cells into endothelial cells, mesenchymal stem cells, and osteoblasts

The controls (ERM cells), Pro-DSLCs, and DSLCs were cultured (0.8 × 10^6^ cells/ml) with the EGM-2 BulletKit (Lonza, Verviers, Belgium) for ECs differentiation (1 week), MSCGM BulletKit (Lonza) for MSCs differentiation (1 week), and RPMI 1640 Medium (Gibco, Thermo Fisher Scientific) with Osteoblast-Inducer Reagent (Takara Bio, Shiga, Japan) for osteogenic differentiation (3 weeks) at 37 °C in an incubator supplied with 5% CO_2_. The mRNA expression levels of the endothelial cell markers *Cd31*, *Cd34*, and *Cd144*, mesenchymal stem cell-negative marker *Cd45*, mesenchymal stem cell-positive markers *Cd90*, and *Cd105*; osteogenic markers *Col1a2*, *Opn*, and *Oc*, and ameloblastic marker *Amel* were evaluated. ECs (Cosmo Bio, Tokyo, Japan) and MSCs (Cell Biologics, IL, USA) were used as positive controls.

The expression levels of the protein markers for osteogenic differentiation were also confirmed. The controls (ERM cells), Pro-DSLCs, and DSLCs were cultured in RPMI 1640 Medium with Osteoblast-Inducer Reagent for 3 weeks and filtered using a FALCON Cell Strainer (100 μm; Corning). The cells were incubated in the dark for 30 min at 4 °C with Alexa Fluor 488 mouse anti-Human alkaline phosphatase (BD Biosciences) and PE mouse anti-human osteocalcin (BD Biosciences), followed by staining with 7-AAD (BD Biosciences). Alkaline phosphatase (ALP) and OC staining in the cells were analyzed by flow cytometry (BD FACSAriaIIIu, BD Biosciences). Furthermore, the controls (ERM cells), Pro-DSLCs, DSLCs, and MSCs were cultured in RPMI 1640 Medium with Osteoblast-Inducer Reagent in a 24-well plate (IWAKI, Tokyo, Japan) for 3 weeks. Subsequently, the calcification levels were examined using the Alizarin Red staining set (PG Research, Tokyo, Japan), and absorbance at 450 nm was determined using a microplate reader.

### Statistical analysis

The experiments were performed in quintuplicate, except for qPSQ (quadruplicate). Data were analyzed using Kruskal–Wallis or χ^2^ tests, with a *p* < 0.01 or < 0.05 considered as significant. The data are presented as means ± standard error. All statistical analyses were performed using SPSS version 23 (IBM, NY, USA).

## Supplementary information

Supplementary information 1.

## Data Availability

The datasets generated during and/or analyzed during the current study are available from the corresponding author on reasonable request.
