# Supplementary material for: Direct reprogramming of epithelial cell rests of malassez into mesenchymal-like cells by epigenetic agents
Source: Sci Rep. 2021 Jan 20;11:1852. doi: 10.1038/s41598-020-79426-4 (PMC7817677; doi:10.1038/s41598-020-79426-4)
Supplement: Supplementary file 1 — Supplementary information 1. [file 41598_2020_79426_MOESM1_ESM.docx]

**Original article**

Direct Reprogramming of Epithelial cell rests of Malassez into mesenchymal-like cells by epigenetic agents

Koki Yoshida^1^, D.D.S., Ph.D., Osamu Uehara^2^, D.D.S., Ph.D., Yoshihito Kurashige^3^, D.D.S., Ph.D., Durga Paudel^1^, D.D.S., Aya Onishi^1^, D.D.S., Ph.D., Puja Neopane^1^, M.S_C_., Ph.D., Daichi Hiraki^4^, D.D.S., Ph.D., Tetsuro Morikawa^1^, D.D.S., Ph.D., Fumiya Harada^5^, D.D.S., Ph.D., Rie Takai^6^, D.D.S., Ph.D., Jun Sato^1^, D.D.S., Ph.D., Masato Saitoh^3^, D.D.S., Ph.D., Yoshihiro Abiko^1^, B.A., D.D.S., Ph.D.

^1^Division of Oral Medicine and Pathology, Department of Human Biology and Pathophysiology, School of Dentistry, Health Sciences University of Hokkaido, 1757 Kanazawa, Ishikari-Tobetsu, Hokkaido, 061-0293, Japan

^2^Division of Disease Control and Molecular Epidemiology, Department of Oral Growth and Development, School of Dentistry, Health Sciences University of Hokkaido, 1757 Kanazawa, Ishikari-Tobetsu, Hokkaido, 061-0293, Japan

^3^Division of Pediatric Dentistry, School of Dentistry, Health Sciences University of Hokkaido, 1757 Kanazawa, Ishikari-Tobetsu, Hokkaido, 061-0293, Japan

^4^Division of Reconstructive Surgery for Oral and Maxillofacial Region, Department of Human Biology and Pathophysiology, School of Dentistry, Health Sciences University of Hokkaido, 1757 Kanazawa, Ishikari-Tobetsu, Hokkaido, 061-0293, Japan

^5^Division of Oral and Maxillofacial Surgery, Department of Human Biology and Pathophysiology, School of Dentistry, Health Sciences University of Hokkaido, 1757 Kanazawa, Ishikari-Tobetsu, Hokkaido, 061-0293, Japan

^6^Research Institute of Health Sciences, Health Sciences University of Hokkaido, 1757 Kanazawa, Ishikari-Tobetsu, Hokkaido, 061-0293, Japan.

**Supplementary Figure S1.** Pyrogram of Pyrosequencing (PSQ). In order to verify the reliability of the MSP data, DNA methylation analysis was performed at the CpG sites using the quantitative PSQ method. The DNA methylation percentage levels of the CpG sites in *Oct3/4* and *Sox2* in the cells treated with 5Aza alone (5Aza1w, and 5Aza2w), Pro-DSLCs, and DSLCs were significantly lower than those in the controls (ERM cells) (***p* < 0.01; χ^2^ tests, n = 4).


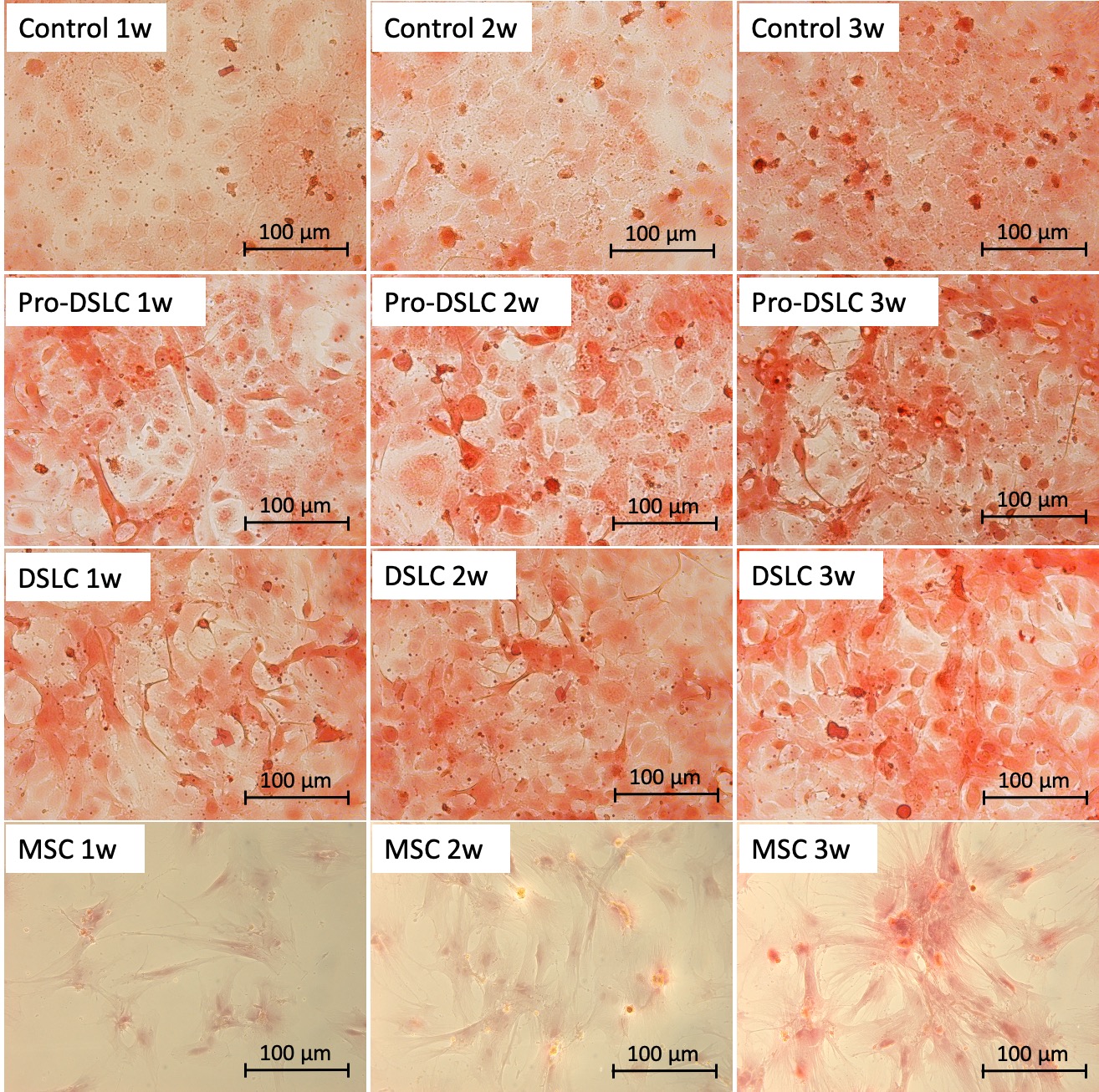


**Supplementary Figure S2.** Phase-contrast microscopy of the cells visualized by Alizarin Red staining. The controls, Pro-DSLCs, DSLCs, and MSCs were cultured in osteogenic differentiation culture medium for 3 weeks. The Pro-DSLCs, DSLC, and MSCs showed increased positive staining for Alizarin Red in a time-dependent manner. Scale bar = 100 μm. Magnification × 400.


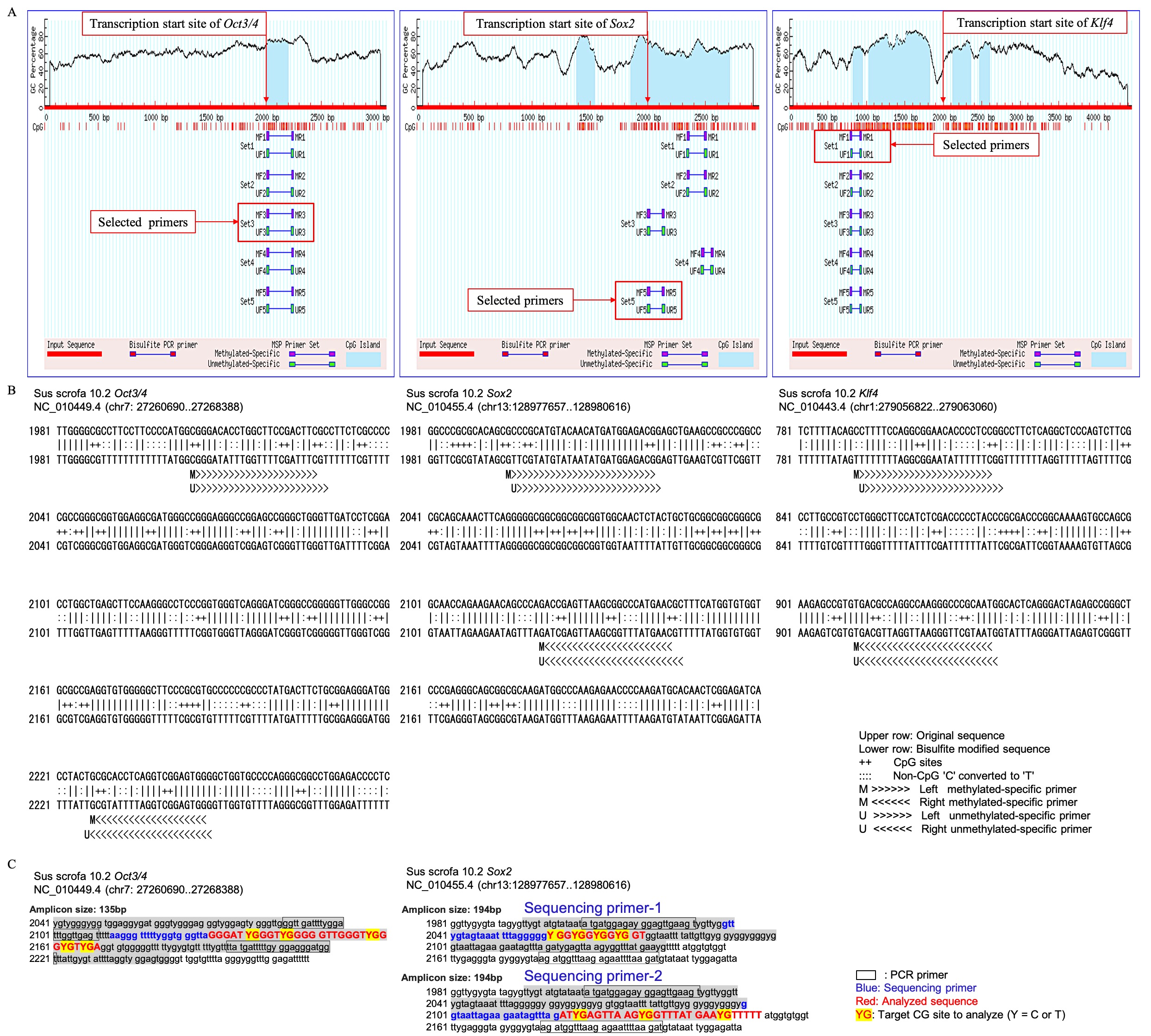


**Supplementary Figure S3.** The designs of the qMSP, and qPSQ primers using MethPrimer. Diagram showing the transcription start site (2000 base pairs), CpG islands, and locations of the selected primers (A). Text showing sequence alignment and location of the primers (B). The qPSQ primers of Oct3/4 and Sox2 were designed in the sequences of the MSP primers (C).
